# The “Adjacent Possible” and Cardiac Innovation

**DOI:** 10.1016/j.jacadv.2022.100162

**Published:** 2022-12-30

**Authors:** Mohamad Alkhouli, Candice Silversides

**Figure:**
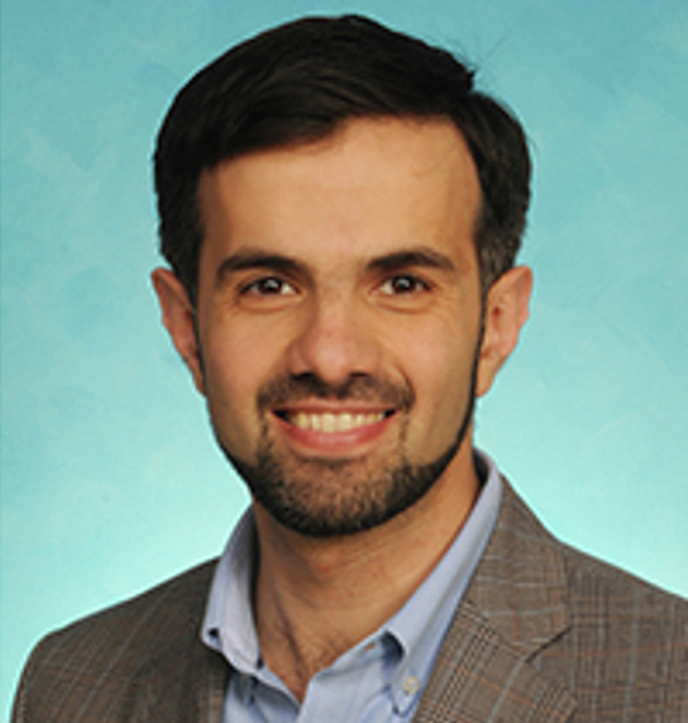

**Figure:**
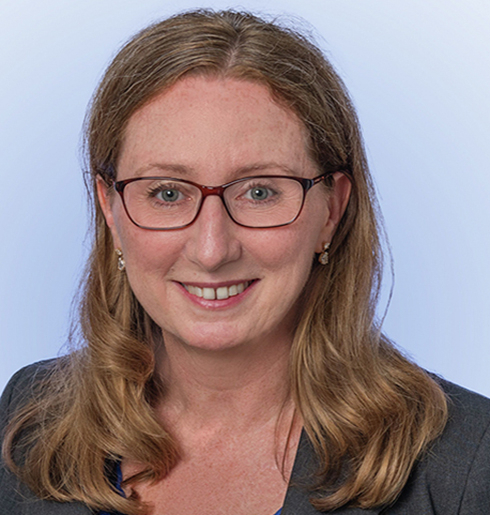

“The adjacent possible is a kind of shadow future, hovering on the edges of the present state of things, a map of all the ways in which the present can reinvent itself.”^1^

The “adjacent possible” is a concept first introduced in evolutionary biology and complex adaptive systems by Dr Stuart Kauffman and subsequently popularized by Steven Johnson in his book “Where good ideas come from: The Natural History of Innovation.”^1^ The concept proposes that the development of complex systems, in nature, culture, science, or technology, occurs by incremental changes based on sets of possible options. Each incremental change leads to new possibilities for future innovations.

In his book, Steven Johnson provides examples of the evolution and the limits of the adjacent possible. In prebiotic earth, he explains, carbon atoms could not spontaneously arrange themselves into complex life forms like a sunflower. They first had to arrange themselves into simpler structures like molecules, proteins, and cells. These first-order combinations are termed “the adjacent possible,” and each step forward results in new possibilities, until, eventually, a carbon atom can become part of a more complex structure like a sunflower. However, great leaps beyond the adjacent possible will often fail because the environment is simply not ready. Steven Johnson explains how YouTube would have failed if launched in the 1990s as fast internet connections for downloading videos and software programs for viewing videos were not available on home computers.^1^ Similarly, had left atrial appendage occlusion (LAAO) devices been introduced in the 1990s, they would have likely flopped as device technology, interventional skillsets, cardiac imaging, and the cardiology community in general were not there yet.

In this issue of *JACC: Advances*, Alkhouli et al^2^ describe the progressive developments, the “adjacent possible,” that lead to the development of the LAAO devices in their state-of-the-art paper titled “Left Atrial Appendage Occlusion: Current Advances and Remaining Challenges.”

The concept that the left atrial appendage (LAA) was a nidus for thrombus in patients with nonvalvular atrial fibrillation (NVAF) and that LAA exclusion could be a therapeutic alternative for stroke prevention goes back to 1949 when Madden reported on the resection of the LAA in 2 patients.^3^ Like many new ideas, the concept required an incubation period before being more widely adopted. By the late 1990s, in part due to the growth of transesophageal echocardiography and an increasing number of studies reporting a high prevalence of LAA thrombus in stroke patients with NVAF, there was renewed interest in the condition.^4^ In addition, while treatment with vitamin K antagonists or direct oral anticoagulants provided excellent stroke prevention, there was growing appreciation of the considerable proportion of the population who had contraindications to anticoagulation. Hence, alternatives strategies were needed.^5^ LAA resection, ligation, or clipping could be performed at the time of cardiac surgeries, but only a small proportion of NVAF patients undergo cardiac surgeries. By the early 2000s, more than half a century after that original report by Madden, advances in technology and the growing unmet clinical needs inspired the pioneering of percutaneous LAAO devices. Engineers and cardiologists had been creating other percutaneous devices and had the skillset and materials necessary to design more complex devices. The interventional community had also become skilled at percutaneous implantation of atrial septal closure devices and percutaneous balloon valvuloplasty and started to experiment with transcatheter valve repair and replacement technologies. Finally, the cardiology community had also grown familiar to the expanding portfolio of percutaneous approaches to the treatment of structural problems.

Since the introduction of the first percutaneous LAA transcatheter occluder (PLATTO) device, many new possibilities have emerged. The new LAAO devices are made with a better material and more effective designs. Cardiac computed tomography and computational modeling can now be used to improve device implantation and decrease peridevice leaks. Improvements in imaging technology and the introduction of 3D intracardiac echocardiography have led to the possibility of contract- and general-anesthesia-free implantations. That adjacent possible, that kind of shadow future, has paved the way for cardiac innovation. With any innovation, tripwires emerge, and the case of LAAO is no exception. Tackling the remaining challenges with LAAO requires concerted efforts to make the train of innovation in stroke prevention safer, faster, and more elegant.
